# Supplementary material for: CryoEM and AI reveal a structure of SARS-CoV-2 Nsp2, a multifunctional protein involved in key host processes
Source: Res Sq. 2021 May 19:rs.3.rs-515215. Preprint. [Version 1] doi: 10.21203/rs.3.rs-515215/v1 (PMC8142659; doi:10.21203/rs.3.rs-515215/v1)
Supplement: Supplement 1 [file e6a17a68e1577becb84a835f.pdf]

Preliminary Full wwPDB EM Validation Report (i)

Feb 3, 2021 – 06:35 PM EST

Deposition ID : D_1000254629

This is a Preliminary Full wwPDB EM Validation Report.

This report is produced by the wwPDB Deposition System during initial deposition but before annotation of the structure.

We welcome your comments at *validation@mail.wwpdb.org*
A user guide is available at
https://www.wwpdb.org/validation/2017/EMValidationReportHelp
with specific help available everywhere you see the (i) symbol.

PRELIMINARY VALIDATION REPORT

The following versions of software and data (see references (i)) were used in the production of this report:

EMDB validation analysis	:	0.0.0.dev61
MolProbity	:	4.02b-467
Percentile statistics	:	20191225.v01 (using entries in the PDB archive December 25th 2019)
Ideal geometry (proteins)	:	Engh & Huber (2001)
Ideal geometry (DNA, RNA)	:	Parkinson et al. (1996)
Validation Pipeline (wwPDB-VP)	:	2.16

1 Overall quality at a glance (i)

The following experimental techniques were used to determine the structure:
ELECTRON MICROSCOPY

The reported resolution of this entry is unknown.

Percentile scores (ranging between 0-100) for global validation metrics of the entry are shown in the following graphic. The table shows the number of entries on which the scores are based.

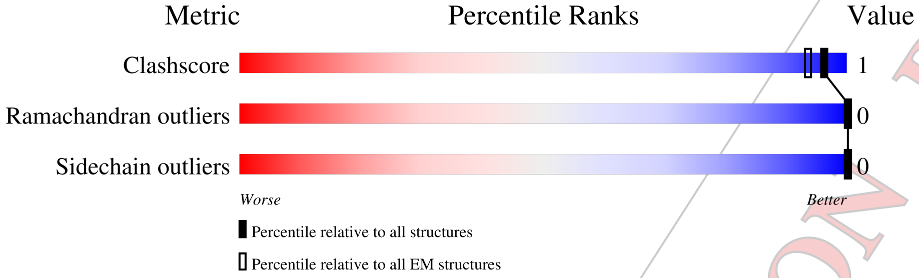

Metric	Whole archive (#Entries)	EM structures (#Entries)
Clashscore	158937	4297
Ramachandran outliers	154571	4023
Sidechain outliers	154315	3826

The table below summarises the geometric issues observed across the polymeric chains and their fit to the map. The red, orange, yellow and green segments of the bar indicate the fraction of residues that contain outliers for >=3, 2, 1 and 0 types of geometric quality criteria respectively. A grey segment represents the fraction of residues that are not modelled. The numeric value for each fraction is indicated below the corresponding segment, with a dot representing fractions <=5% The upper red bar (where present) indicates the fraction of residues that have poor fit to the EM map (all-atom inclusion < 40%). The numeric value is given above the bar.

Mol	Chain	Length	Quality of chain
1	A	638	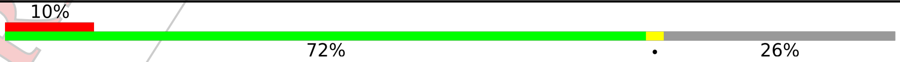

PRELIMINARY VALIDATION REPORT

2 Entry composition (i)

There are 2 unique types of molecules in this entry. The entry contains 3706 atoms, of which 0 are hydrogens and 0 are deuteriums.

In the tables below, the AltConf column contains the number of residues with at least one atom in alternate conformation and the Trace column contains the number of residues modelled with at most 2 atoms.

- Molecule 1 is a protein called Chains: A.

Mol	Chain	Residues	Atoms					AltConf	Trace
			Total	C	N	O	S		
1	A	473	3703	2359	620	698	26	0	0

- Molecule 2 is ZINC ION (three-letter code: ZN) (formula: Zn).

Mol	Chain	Residues	Atoms		AltConf
			Total	Zn	
2	B	1	3	3	0
2	B	1	3	3	0
2	B	1	3	3	0

PRELIMINARY VALIDATION REPORT

3 Residue-property plots ⓘ

These plots are drawn for all protein, RNA, DNA and oligosaccharide chains in the entry. The first graphic for a chain summarises the proportions of the various outlier classes displayed in the second graphic. The second graphic shows the sequence view annotated by issues in geometry and atom inclusion in map density. Residues are color-coded according to the number of geometric quality criteria for which they contain at least one outlier: green = 0, yellow = 1, orange = 2 and red = 3 or more. A red diamond above a residue indicates a poor fit to the EM map for this residue (all-atom inclusion < 40%). Stretches of 2 or more consecutive residues without any outlier are shown as a green connector. Residues present in the sample, but not in the model, are shown in grey.

- Molecule 1: Chains: A

Chain A:

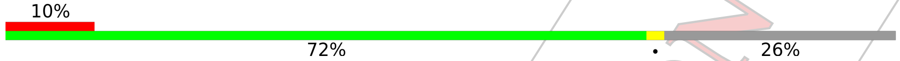

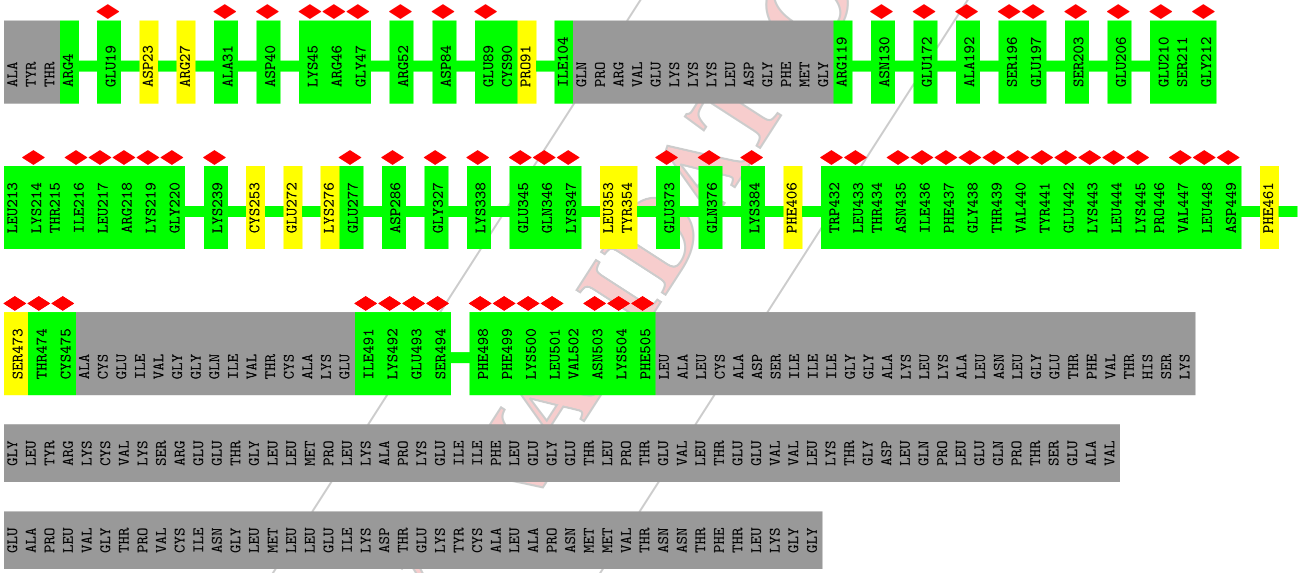

PRELIMINARY VALIDATION REPORT

4 Experimental information (i)

Property	Value	Source
EM reconstruction method	SINGLE PARTICLE	Depositor
Imposed symmetry	POINT, Not provided	
Number of particles used	Not provided	
Resolution determination method	Not provided	
CTF correction method	Not provided	
Microscope	Not provided	
Voltage (kV)	Not provided	
Electron dose $(e^-/\text{Å}^2)$	Not provided	
Minimum defocus (nm)	Not provided	
Maximum defocus (nm)	Not provided	
Magnification	Not provided	
Image detector	Not provided	
Maximum map value	0.157	Depositor
Minimum map value	-0.088	Depositor
Average map value	-0.000	Depositor
Map value standard deviation	0.003	Depositor
Recommended contour level	0.032	Depositor
Map size (Å)	233.52, 233.52, 233.52	wwPDB
Map dimensions	280, 280, 280	wwPDB
Map angles (°)	90.0, 90.0, 90.0	wwPDB
Pixel spacing (Å)	0.834, 0.834, 0.834	Depositor

PRELIMINARY VALIDATION REPORT

5 Model quality (i)

5.1 Standard geometry (i)

Bond lengths and bond angles in the following residue types are not validated in this section: ZN

The Z score for a bond length (or angle) is the number of standard deviations the observed value is removed from the expected value. A bond length (or angle) with $|Z| > 5$ is considered an outlier worth inspection. RMSZ is the root-mean-square of all Z scores of the bond lengths (or angles).

Mol	Chain	Bond lengths		Bond angles	
		RMSZ	#$|Z|$ >5	RMSZ	#$|Z|$ >5
1	A	0.63	1/3777 (0.0%)	0.47	0/5103

All (1) bond length outliers are listed below:

Mol	Chain	Res	Type	Atoms	Z	Observed(Å)	Ideal(Å)
1	A	91	PRO	N-CD	-5.30	1.40	1.47

There are no bond angle outliers.

There are no chirality outliers.

There are no planarity outliers.

5.2 Too-close contacts (i)

In the following table, the Non-H and H(model) columns list the number of non-hydrogen atoms and hydrogen atoms in the chain respectively. The H(added) column lists the number of hydrogen atoms added and optimized by MolProbity. The Clashes column lists the number of clashes within the asymmetric unit, whereas Symm-Clashes lists symmetry-related clashes.

Mol	Chain	Non-H	H(model)	H(added)	Clashes	Symm-Clashes
1	A	3703	0	3667	6	0
2	B	3	0	0	0	0
All	All	3706	0	3667	6	0

The all-atom clashscore is defined as the number of clashes found per 1000 atoms (including hydrogen atoms). The all-atom clashscore for this structure is 1.

All (6) close contacts within the same asymmetric unit are listed below, sorted by their clash magnitude.

PRELIMINARY VALIDATION REPORT

Atom-1	Atom-2	Interatomic distance (Å)	Clash overlap (Å)
1:A:272:GLU:O	1:A:276:LYS:NZ	2.39	0.56
1:A:353:LEU:O	1:A:354:TYR:HB2	2.13	0.47
1:A:23:ASP:OD2	1:A:27:ARG:NH1	2.48	0.47
1:A:461:PHE:CG	1:A:461:PHE:O	2.72	0.43
1:A:253:CYS:SG	1:A:253:CYS:O	2.77	0.42
1:A:406:PHE:HD1	1:A:473:SER:HG	1.68	0.42

There are no symmetry-related clashes.

5.3 Torsion angles (i)

5.3.1 Protein backbone (i)

In the following table, the Percentiles column shows the percent Ramachandran outliers of the chain as a percentile score with respect to all PDB entries followed by that with respect to all EM entries.

The Analysed column shows the number of residues for which the backbone conformation was analysed, and the total number of residues.

Mol	Chain	Analysed	Favoured	Allowed	Outliers	Percentiles	
1	A	467/638 (73%)	458 (98%)	9 (2%)	0	100	100

There are no Ramachandran outliers to report.

5.3.2 Protein sidechains (i)

In the following table, the Percentiles column shows the percent sidechain outliers of the chain as a percentile score with respect to all PDB entries followed by that with respect to all EM entries.

The Analysed column shows the number of residues for which the sidechain conformation was analysed, and the total number of residues.

Mol	Chain	Analysed	Rotameric	Outliers	Percentiles	
1	A	407/545 (75%)	407 (100%)	0	100	100

There are no protein residues with a non-rotameric sidechain to report.

Sometimes sidechains can be flipped to improve hydrogen bonding and reduce clashes. There are no such sidechains identified.

PRELIMINARY VALIDATION REPORT

5.3.3 RNA (i)

There are no RNA molecules in this entry.

5.4 Non-standard residues in protein, DNA, RNA chains (i)

There are no non-standard protein/DNA/RNA residues in this entry.

5.5 Carbohydrates (i)

There are no monosaccharides in this entry.

5.6 Ligand geometry (i)

Of 3 ligands modelled in this entry, 3 are monoatomic - leaving 0 for Mogul analysis.

There are no bond length outliers.

There are no bond angle outliers.

There are no chirality outliers.

There are no torsion outliers.

There are no ring outliers.

No monomer is involved in short contacts.

5.7 Other polymers (i)

There are no such residues in this entry.

5.8 Polymer linkage issues (i)

There are no chain breaks in this entry.

PRELIMINARY VALIDATION REPORT

6 Map visualisation (i)

This section contains visualisations of the EMDB entry D_1000254629. These allow visual inspection of the internal detail of the map and identification of artifacts.

Images derived from a raw map, generated by summing the deposited half-maps, are presented below the corresponding image components of the primary map to allow further visual inspection and comparison with those of the primary map.

6.1 Orthogonal projections (i)

6.1.1 Primary map

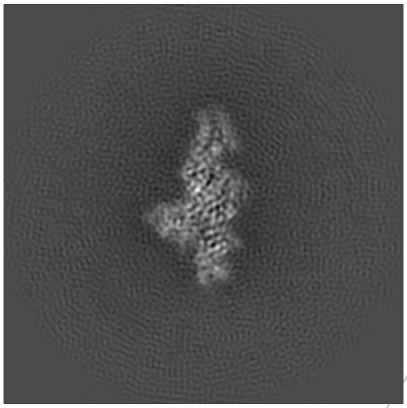
X

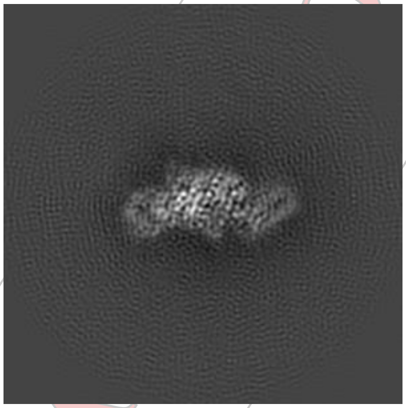
Y

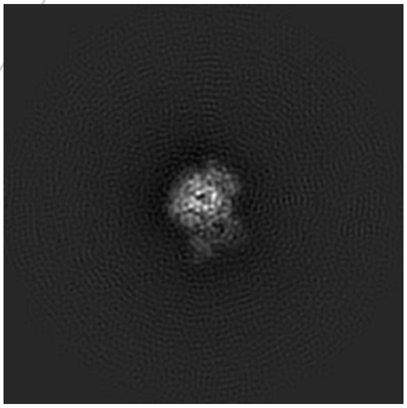
Z

6.1.2 Raw map

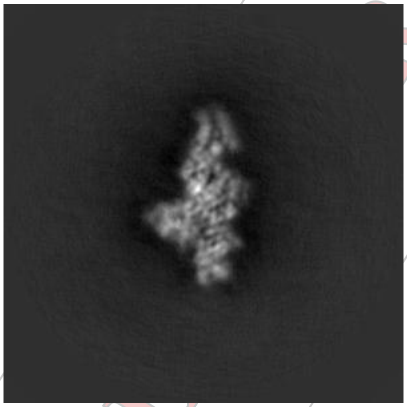
X

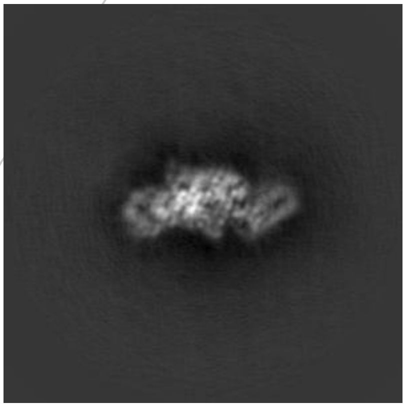
Y

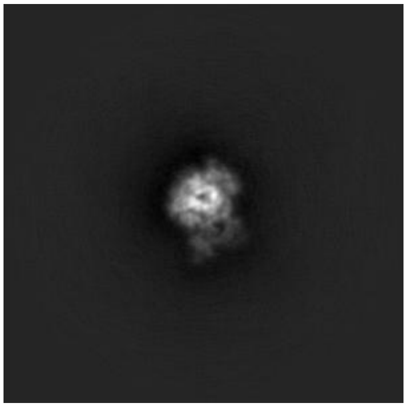
Z

The images above show the map projected in three orthogonal directions.

PRELIMINARY VALIDATION REPORT

6.2 Central slices (i)

6.2.1 Primary map

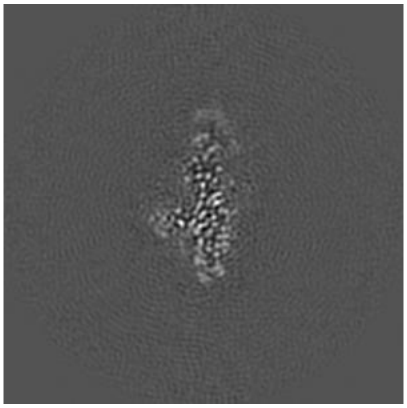

X Index: 140

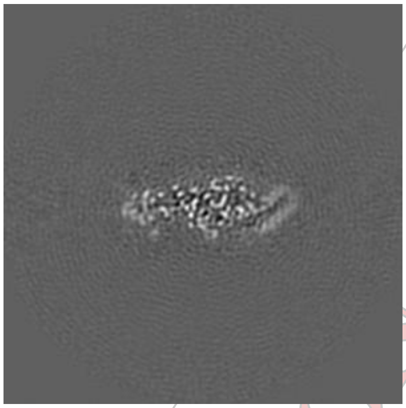

Y Index: 140

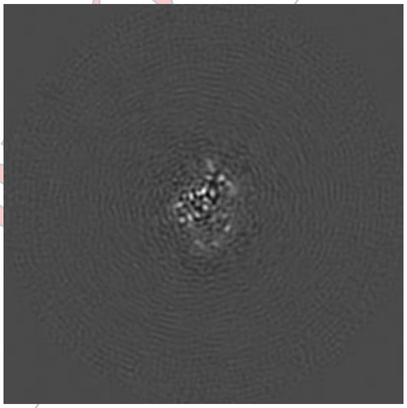

Z Index: 140

6.2.2 Raw map

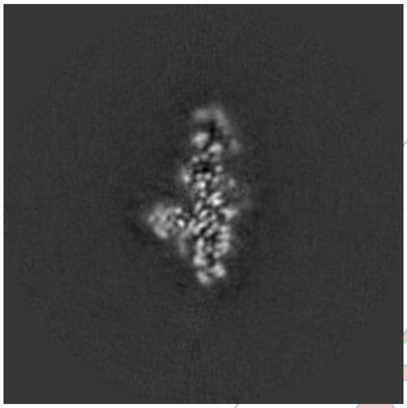

X Index: 140

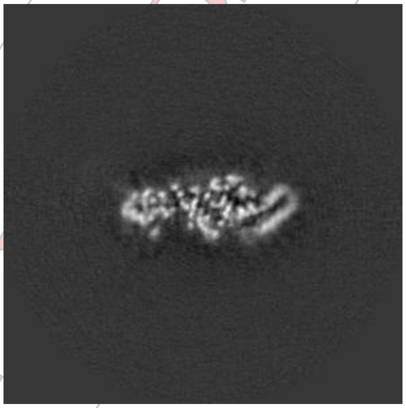

Y Index: 140

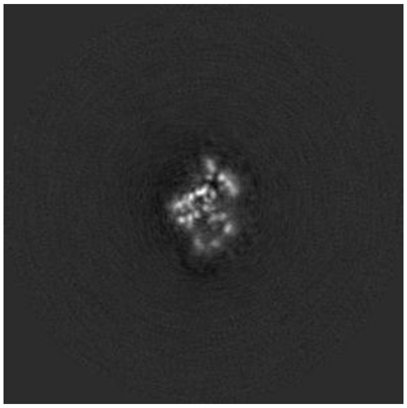

Z Index: 140

The images above show central slices of the map in three orthogonal directions.

PRELIMINARY VALIDATION REPORT

6.3 Largest variance slices (i)

6.3.1 Primary map

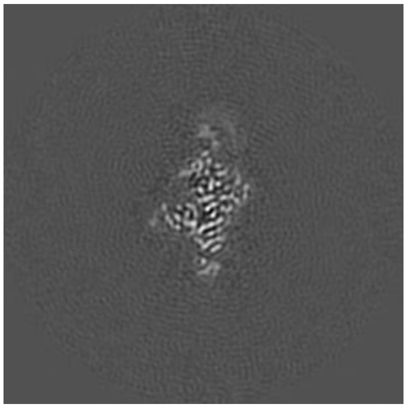
X Index: 146

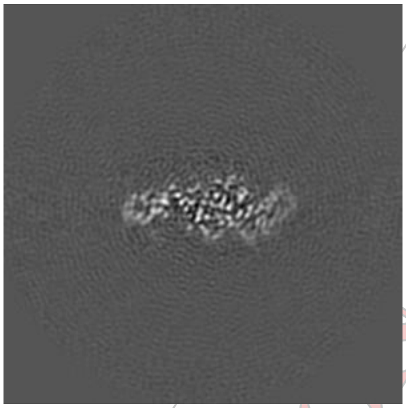
Y Index: 138

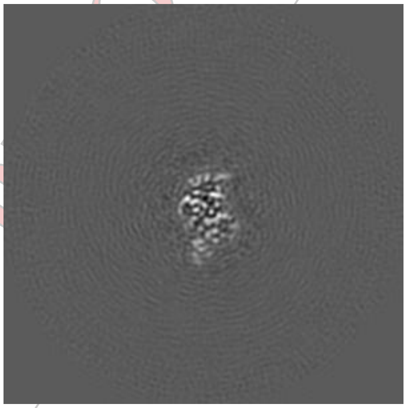
Z Index: 132

6.3.2 Raw map

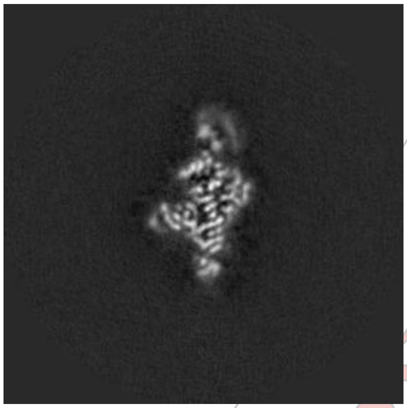
X Index: 146

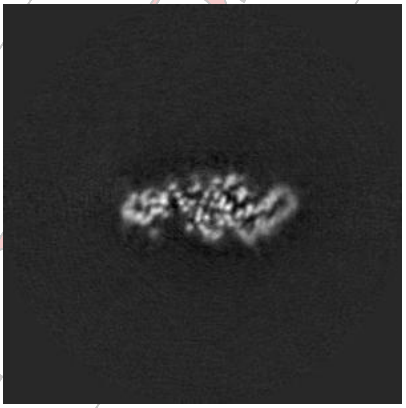
Y Index: 138

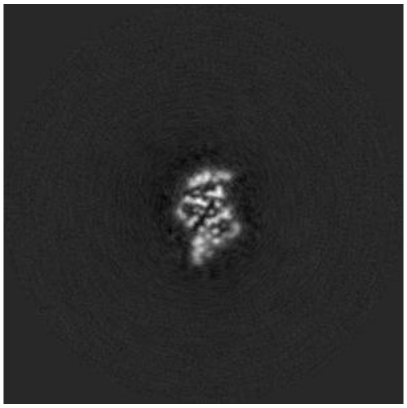
Z Index: 133

The images above show the largest variance slices of the map in three orthogonal directions.

PRELIMINARY VALIDATION REPORT

6.4 Orthogonal surface views (i)

6.4.1 Primary map

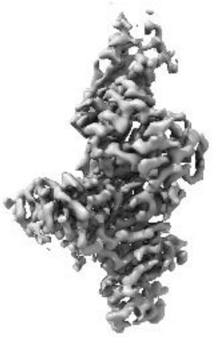

X

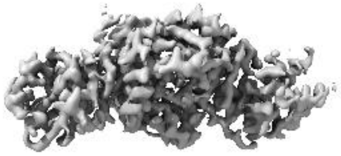

Y

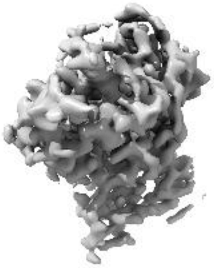

Z

The images above show the 3D surface view of the map at the recommended contour level 0.032. These images, in conjunction with the slice images, may facilitate assessment of whether an appropriate contour level has been provided.

6.4.2 Raw map

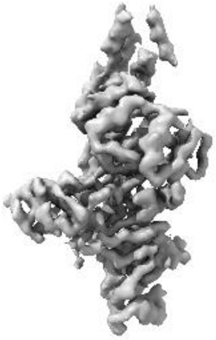

X

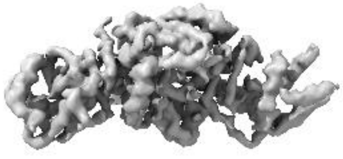

Y

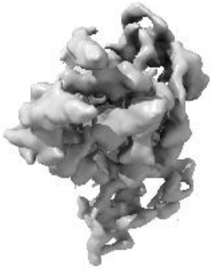

Z

These images show the 3D surface of the raw map. The raw map's contour level was selected so that its surface encloses the same volume as the primary map does at its recommended contour level.

PRELIMINARY VALIDATION REPORT

6.5 Mask visualisation ⓘ

This section shows the 3D surface view of the primary map at 50% transparency overlaid with the specified mask at 0% transparency

A mask typically either:

- Encompasses the whole structure
- Separates out a domain, a functional unit, a monomer or an area of interest from a larger structure

6.5.1 D_1000254629_em-mask-volume_P1.map.V2 ⓘ

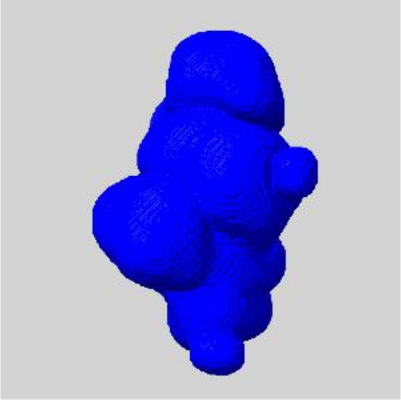

X

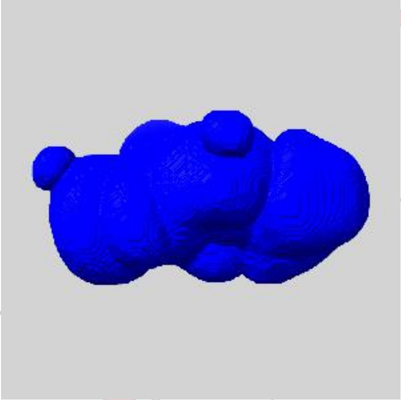

Y

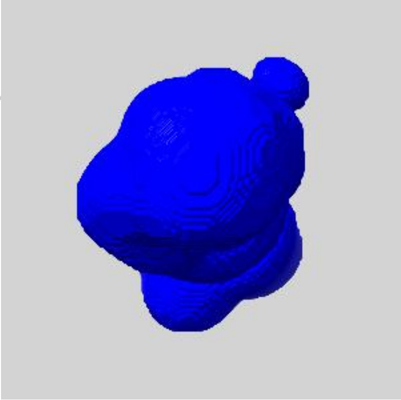

Z

PRELIMINARY VALIDATION REPORT

7 Map analysis (i)

This section contains the results of statistical analysis of the map.

7.1 Map-value distribution (i)

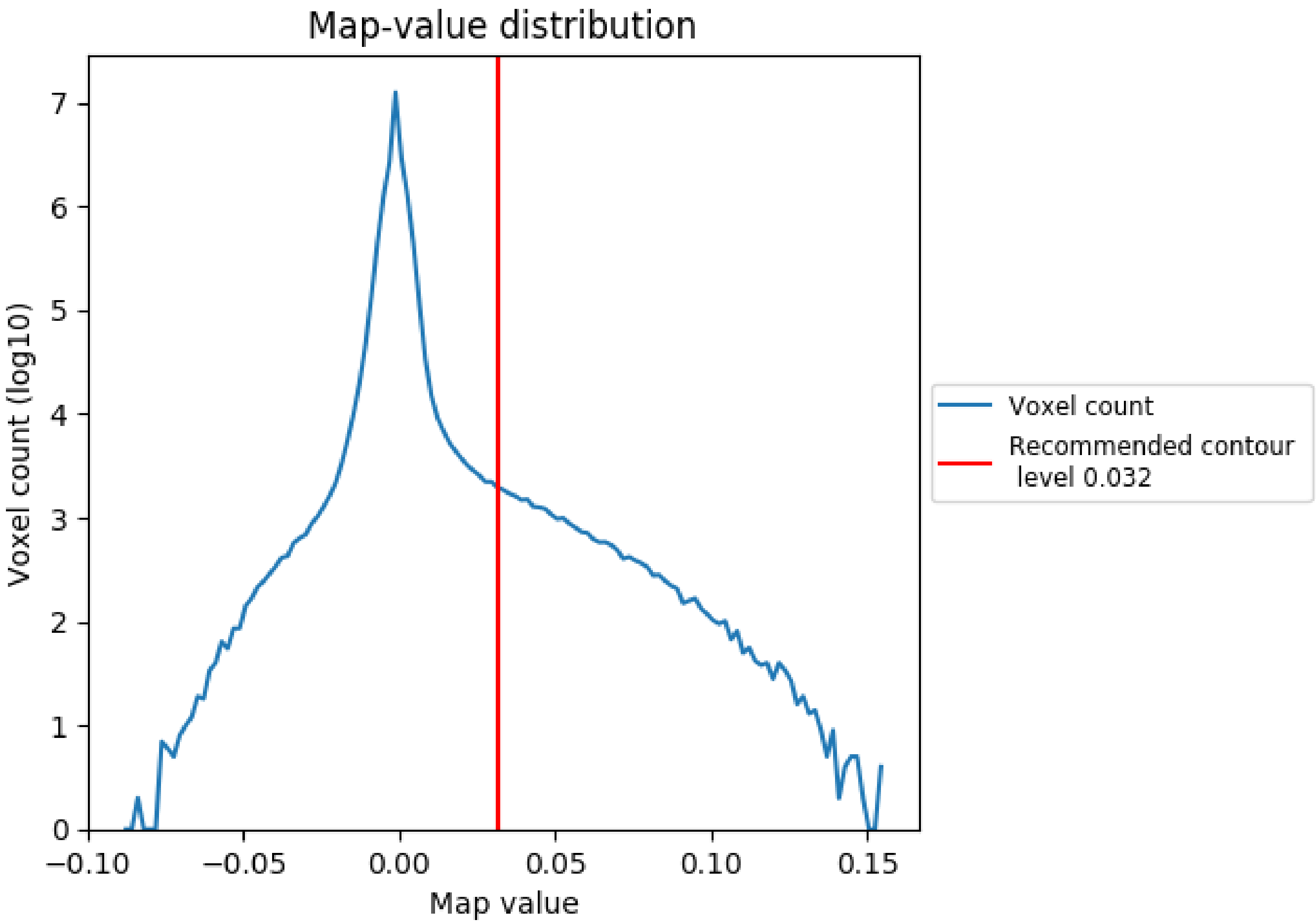

The map-value distribution is plotted in 128 intervals along the x-axis. The y-axis is logarithmic. A spike in this graph at zero usually indicates that the volume has been masked.

PRELIMINARY REPORT

7.2 Volume estimate (i)

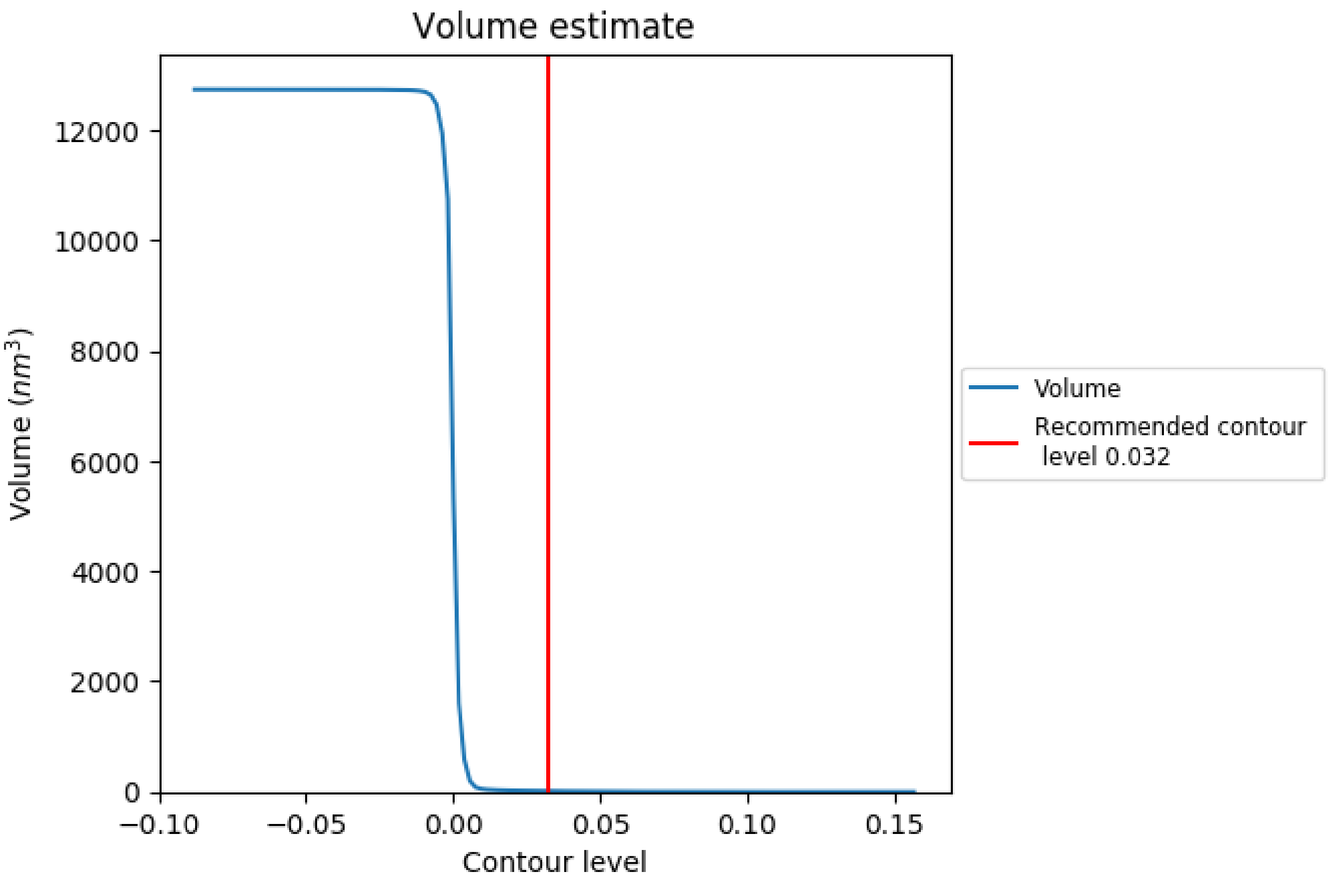

The volume at the recommended contour level is 16 nm^3; this corresponds to an approximate mass of 14 kDa.

The volume estimate graph shows how the enclosed volume varies with the contour level. The recommended contour level is shown as a vertical line and the intersection between the line and the curve gives the volume of the enclosed surface at the given level.

PRELIMINARY VA

7.3 Rotationally averaged power spectrum (i)

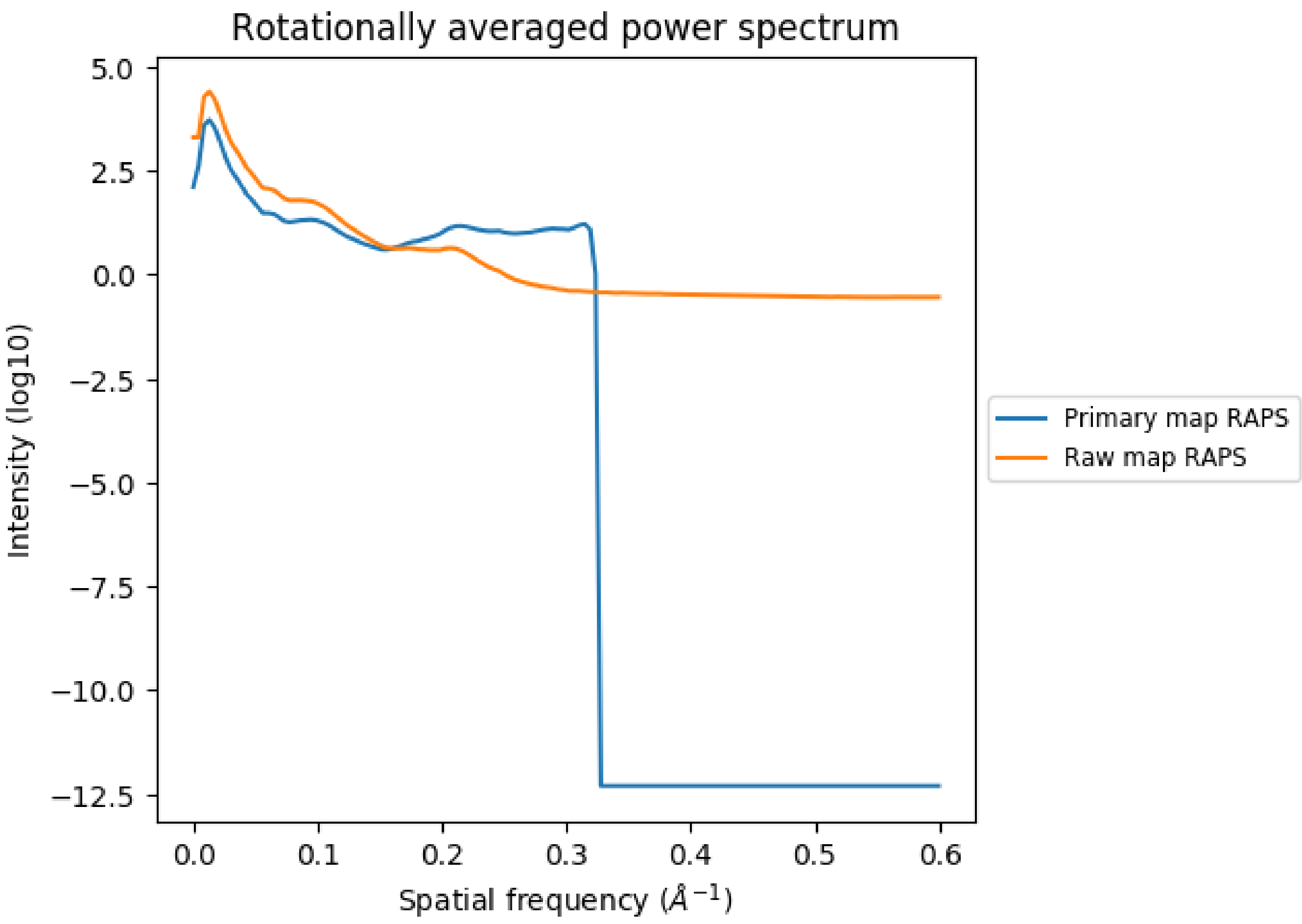

PRELIMINARY

8 Fourier-Shell correlation (i)

Fourier-Shell Correlation (FSC) is the most commonly used method to estimate the resolution of single-particle and subtomogram-averaged maps. The shape of the curve depends on the imposed symmetry, mask and whether or not the two 3D reconstructions used were processed from a common reference. The reported resolution is shown as a black line. A curve is displayed for the half-bit criterion in addition to lines showing the 0.143 gold standard cut-off and 0.5 cut-off.

8.1 FSC (i)

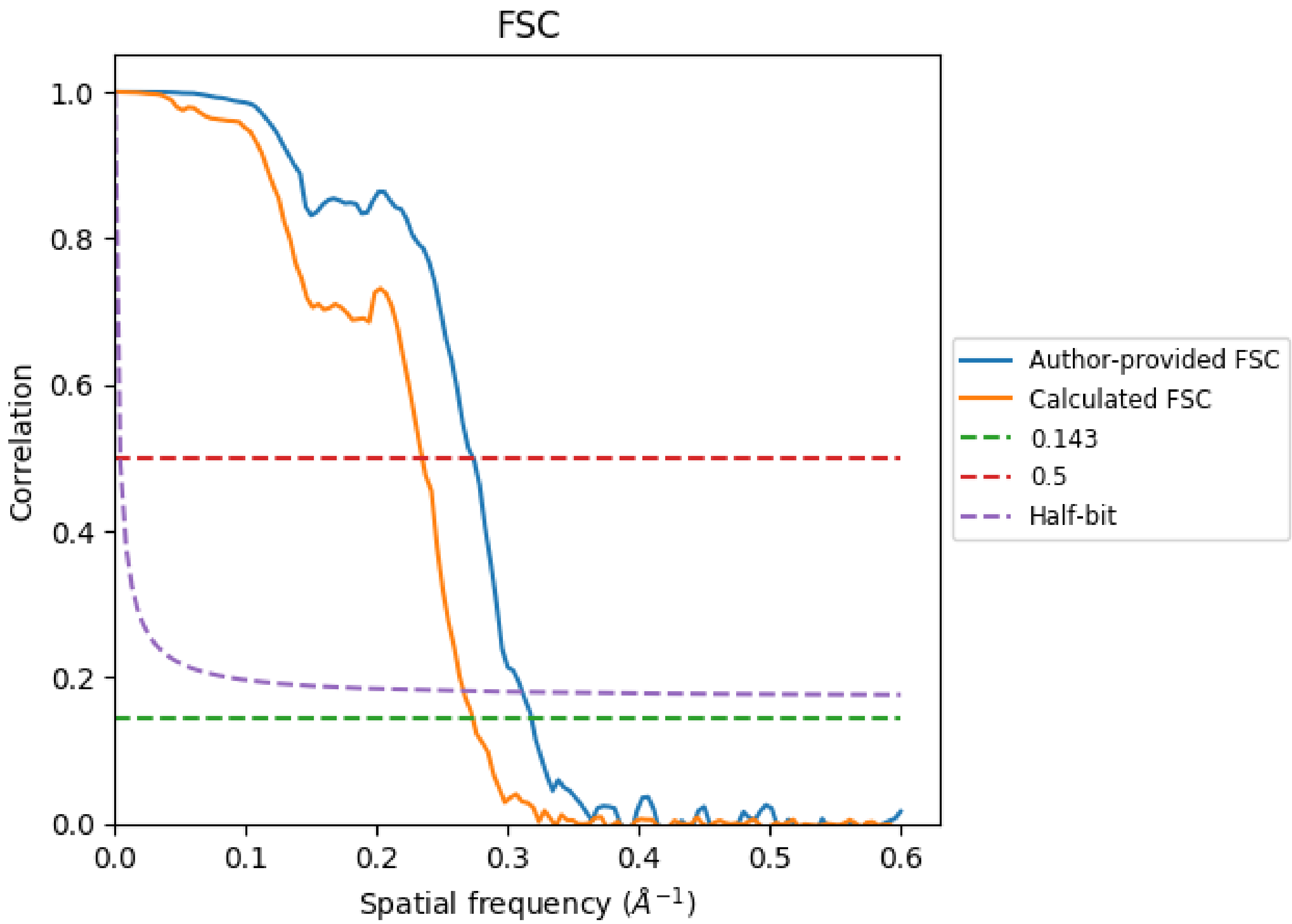

PRELIMINARY REPORT

8.2 Resolution estimates (i)

Resolution estimate (Å)	Estimation criterion (FSC cut-off)		
	0.143	0.5	Half-bit
Reported by author	-	-	-
Author-provided FSC curve	3.19	3.72	3.26
Calculated*	3.72	4.35	3.82

*Resolution estimate based on FSC curve calculated by comparison of deposited half-maps.

PRELIMINARY VALIDATION REPORT

9 Map-model fit (i)

This section contains information regarding the fit between EMDB map D_1000254629 and PDB model D_1000254629. Per-residue inclusion information can be found in section 3 on page 4.

9.1 Map-model overlay (i)

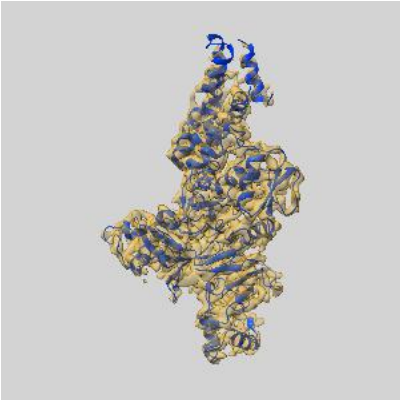

X

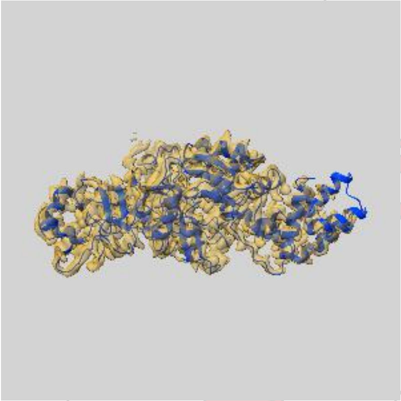

Y

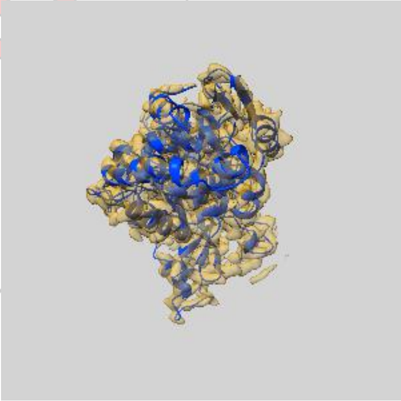

Z

The images above show the 3D surface view of the map at the recommended contour level 0.032 at 50% transparency in yellow overlaid with a ribbon representation of the model coloured in blue. These images allow for the visual assessment of the quality of fit between the atomic model and the map.

PRELIMINARY VALIDATION REPORT

9.2 Atom inclusion (i)

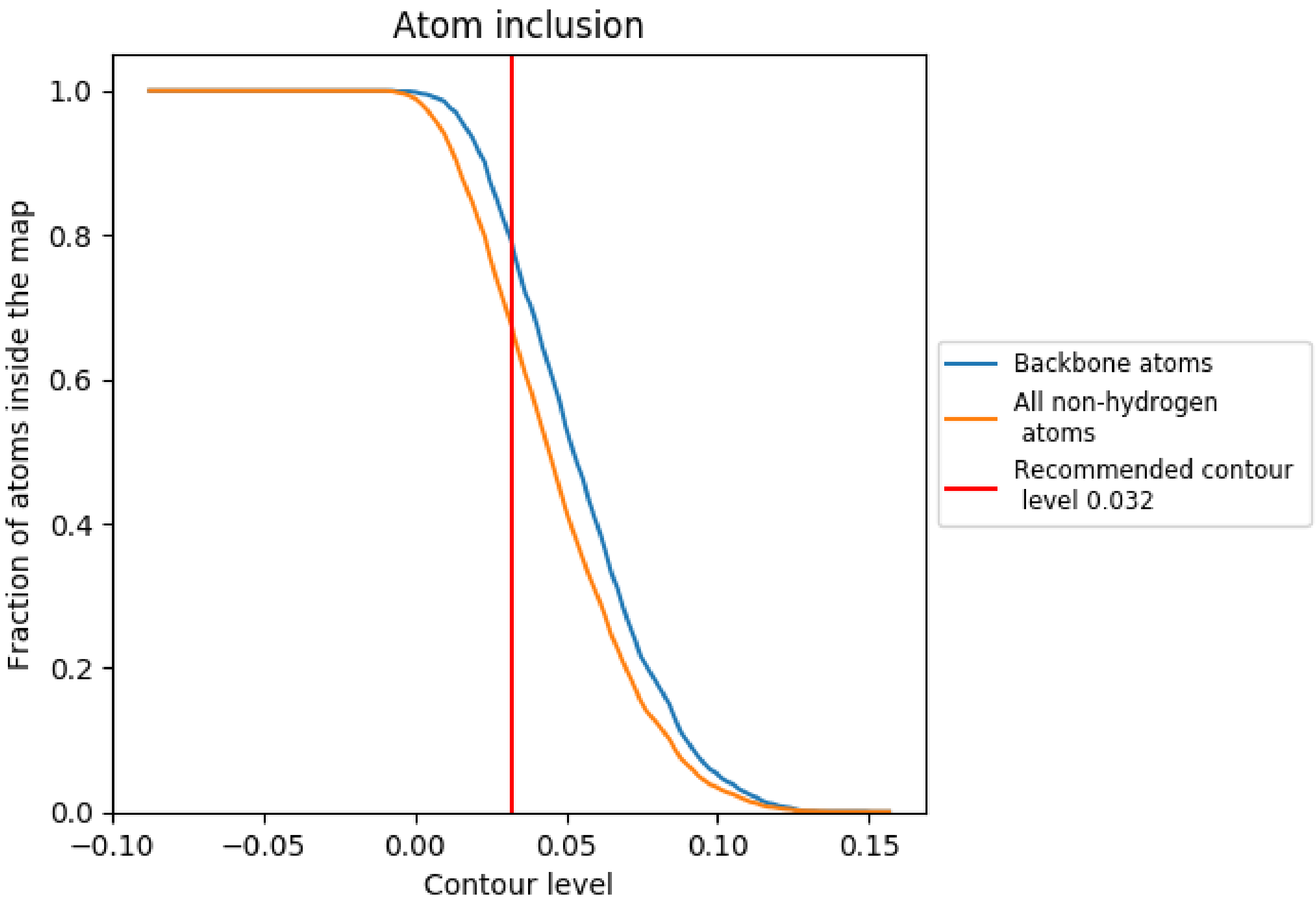

At the recommended contour level, 79% of all backbone atoms, 67% of all non-hydrogen atoms, are inside the map.

PRELIMINARY
